# Urban sports fields support higher levels of soil butyrate and butyrate‐producing bacteria than urban nature parks

**DOI:** 10.1002/ece3.70057

**Published:** 2024-07-22

**Authors:** Joel E. Brame, Craig Liddicoat, Catherine A. Abbott, Christian Cando‐Dumancela, Nicole W. Fickling, Jake M. Robinson, Martin F. Breed

**Affiliations:** ^1^ College of Science and Engineering Flinders University Bedford Park South Australia Australia; ^2^ The Aerobiome Innovation and Research Hub (The AIR Hub), College of Science and Engineering Flinders University Bedford Park South Australia Australia

**Keywords:** butyrate, butyrate‐producing bacteria, molecular ecology, short‐chain fatty acid, soil microbiota, urban greenspaces

## Abstract

Butyrate‐producing bacteria colonise the gut of humans and non‐human animals, where they produce butyrate, a short‐chain fatty acid with known health benefits. Butyrate‐producing bacteria also reside in soils and soil bacteria can drive the assembly of airborne bacterial communities (the aerobiome). Aerobiomes in urban greenspaces are important reservoirs of butyrate‐producing bacteria as they supplement the human microbiome, but soil butyrate producer communities have rarely been examined in detail. Here, we studied soil metagenome taxonomic and functional profiles and soil physicochemical data from two urban greenspace types: sports fields (*n* = 11) and nature parks (*n* = 22). We also developed a novel method to quantify soil butyrate and characterised the in situ activity of butyrate‐producing bacteria. We show that soil butyrate was higher in sports fields than nature parks and that sports fields also had significantly higher relative abundances of the terminal butyrate production genes *buk* and *butCoAT* than nature parks. Soil butyrate positively correlated with *buk* gene abundance (but not *butCoAT*). Soil moisture (*r* = .50), calcium (*r* = −.62), iron (*ρ* = .54), ammonium nitrogen (*ρ* = .58) and organic carbon (*r* = .45) had the strongest soil abiotic effects on soil butyrate concentrations and iron (*ρ* = .56) and calcium (*ρ* = −.57) had the strongest soil abiotic effects on *buk* read abundances. Overall, our findings contribute important new insights into the role of sports fields as key exposure reservoirs of butyrate producing bacteria, with important implications for the provision of microbiome‐mediated human health benefits via butyrate.

## Graphical abstract

Soil bacteria in urban greenspaces influence the assembly of airborne bacterial communities, which can supplement the human microbiome. We compared the abundances of soil butyrate‐producing bacteria and in situ soil butyrate concentrations from two urban greenspace types: sports fields (*n* = 11) and nature parks (*n* = 22). We show that soil butyrate and the abundances of terminal butyrate production genes were higher in sports fields than nature parks and that soil moisture, calcium, iron, ammonium nitrogen and organic carbon had the strongest abiotic effects on soil butyrate concentrations.

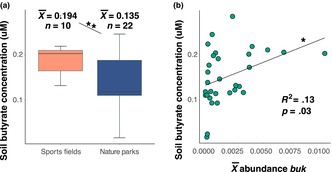

## 1 INTRODUCTION

The environment has a primary role in shaping human commensal bacterial communities (Rothschild et al., 2018). When humans spend time outdoors, environmental microbiota such as air‐ and soil‐borne bacteria can transfer to the body and influence the human microbiome (Roslund et al., 2022; Sessitsch et al., 2023). While much health‐related research has examined transfer dynamics with a focus on mitigating exposure to pathogenic environmental bacteria, outdoor environments can also house bacterial communities with salutogenic characteristics, including diversity that can protect against pathogens (Spragge et al., 2023) and the presence of specific health‐promoting taxonomic groups (e.g., probiotic and butyrate‐producing bacteria; Brame et al., 2022; Roslund et al., 2020; Zhang et al., 2023). Thus, environmental bacteria—particularly soil bacteria—are key sources of the human microbiota and have been demonstrated to play a role in immunoregulation with downstream health implications (Roslund et al., 2020, 2022).

Humans are exposed to soil bacteria via direct contact with soil (Selway et al., 2020) and ingestion of plants with soil residues (Flandroy et al., 2018). These exposures can alter the skin (Grönroos et al., 2019), nose (Selway et al., 2020) and potentially gut bacterial communities (Nurminen et al., 2018). In addition, airborne bacterial communities (aerobiomes) can be inhaled into the respiratory tract and oral surfaces (Flies et al., 2020). Growing evidence shows that exposure to diverse bacteria helps train the adaptive immune system and may regulate the innate immune system, often described as the Biodiversity Hypothesis (Haahtela, 2019). As such, outdoor air and soil are important reservoirs of bacteria to which humans may be exposed via outdoor activities. Therefore, understanding the microbial compositional variation and its ecological drivers in outdoor environments, particularly urban greenspaces, is a critical step in managing environmental microbiome exposure.

The ecology of urban greenspaces influences the composition of the microbial communities in these areas. Urban vegetation influences the diversity and abundance of soil bacteria and fungi (Baruch et al., 2020). Greenspace type and human population density have an effect on the soil microbial community composition (Wang et al., 2018). Furthermore, urban plant–soil systems provide key inputs into aerobiomes (Robinson et al., 2020), as surface bacteria disperse into the air (Bowers et al., 2011). Highly vegetated urban parks have distinct aerobiome compositions from non‐vegetated and nearby parking lots (Mhuireach et al., 2016) and tree density, proximity and canopy coverage modulate urban aerobiome alpha diversity (Robinson et al., 2021). Thus, soils and vegetation influence the greenspace microbial communities to which humans are exposed. As such, rapid urbanisation creates an urgent need to better understand the ecological influences on urban greenspace microbial communities, particularly for specific health‐associated microbiota such as butyrate‐producing bacteria.

Butyrate‐producing bacteria are key human gut taxa with important health implications. Butyrate is produced in anaerobic conditions through a fermentative enzymatic pathway that requires iron and has multiple, sometimes reversible, steps (Vital et al., 2014; Figure S1). Butyrate provides numerous health benefits for humans, including metabolic energy for gut epithelial cells (Rivière et al., 2016), maintenance of gut homeostasis (Parada Venegas et al., 2019) and inhibition of the enzyme histone deacetylase, resulting in epigenetic modifications with anti‐inflammatory and immunoregulatory outcomes such as an increase in regulatory T‐cells (Pandiyan et al., 2019; Sivaprakasam et al., 2017). However, a range of human health conditions are associated with a reduction in gut butyrate‐producing bacteria including asthma (Demirci et al., 2019), atopic dermatitis (Lee et al., 2022) and inflammatory bowel disease (Parada Venegas et al., 2019). Given that the human gut bacterial community is shaped strongly by environmental exposures (Gilbert et al., 2018; Rothschild et al., 2018), urban greenspaces are compelling potential sources of commensal bacteria that could supplement the human microbiome. Thus, there is an unfilled need to further examine how the ecology of urban greenspaces—e.g., soil biotic and abiotic factors, vegetation—influences the abundance and exposure to butyrate‐producing bacteria (Brame et al., 2021).

Here, we investigated the effects of greenspace type and soil abiotic factors on soil butyrate levels and butyrate‐producing bacteria read abundances in greater metropolitan Adelaide, South Australia. We did this by developing a new method to quantify soil butyrate levels and combined this dataset with soil shotgun metagenomic and comprehensive soil physicochemical data. We generated these data from two greenspace types: intensively‐managed grassy sports fields (*n* = 11 sites) and minimally‐managed amenity grassland parks with more natural vegetation systems (*n* = 22 sites). We asked the following research question: what effects do greenspace type and soil abiotic conditions have on soil butyrate concentrations, butyrate‐producing bacteria read abundances and functional abundances of genes for terminal butyrate‐production enzymes? Our study provides a new view on greenspaces by focussing on their soil butyrate and butyrate‐producing bacterial levels.

## 2 MATERIALS AND METHODS

### 2.1 Study sites

**FIGURE 1:**
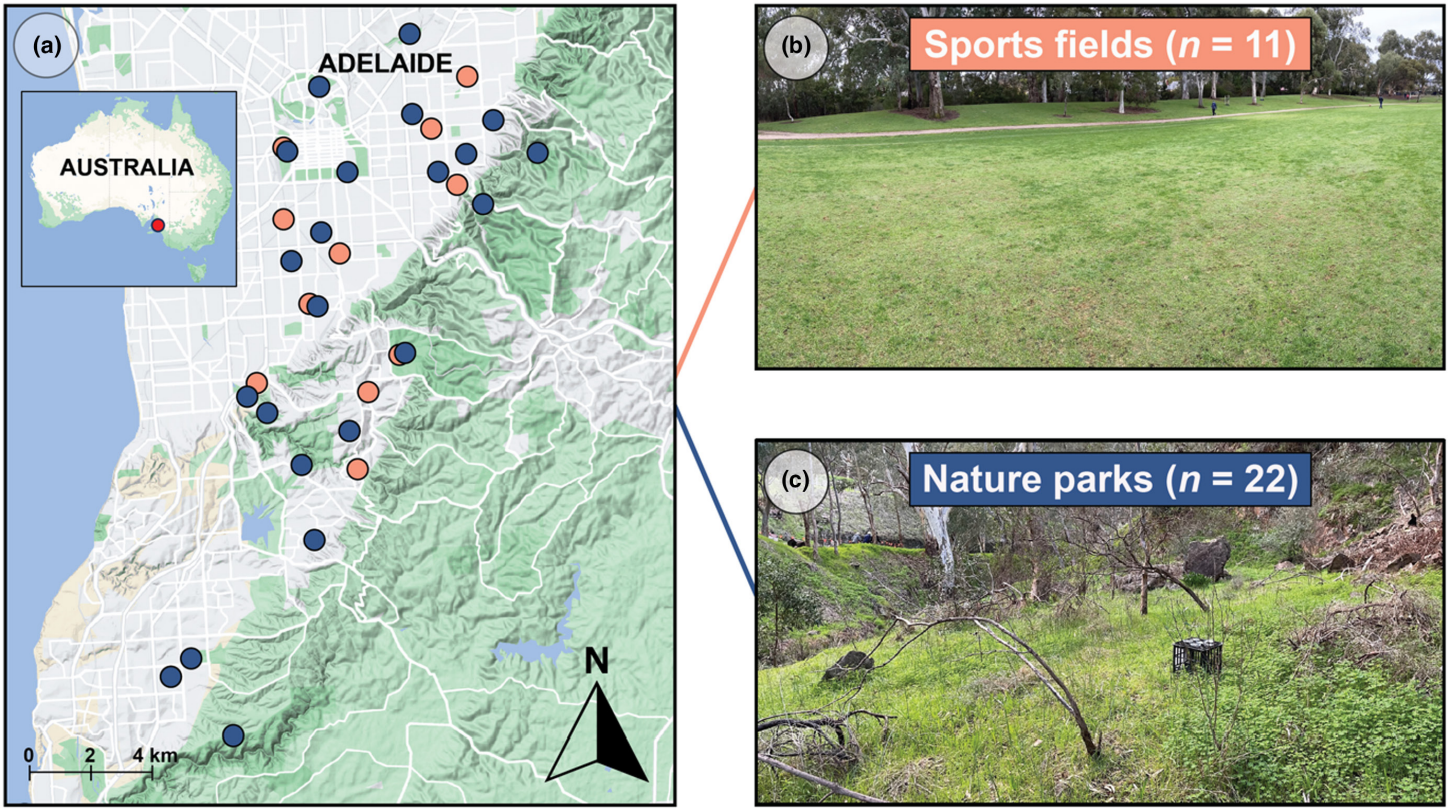
(a) Map showing sample sites across greater metropolitan Adelaide, Australia (orange dots = sports fields, blue dots = nature parks). (b) Representative photo of a sports field site. (c) Representative photo of a nature park site.

We sampled spatially independent sports field (*n* = 11) and nature park (*n* = 22) (33 sites total) sites in greater metropolitan Adelaide, South Australia, using a 25 × 25 m sampling area that is considered appropriate for characterising vegetation and microbial communities at the site‐scale (Baruch et al., 2020; Mills et al., 2020; Figure 1a–c). Sites were chosen so that: (Rothschild et al., 2018) all sites were >5 km from the coast to avoid coastal effects; (Sessitsch et al., 2023) all sites were within the low‐elevation metropolitan Adelaide plains to minimise climatic variation across sites; and (Roslund et al., 2022) nature park sites represented a range of woody vegetation complexity.

### 2.2 Greenspace type classification

We utilised woody species diversity to classify our sites into greenspace types as sports fields or nature parks. We surveyed vegetation at all sites between August 14 and 25, 2022, using established methods from White et al. (2012). In brief, this included assessing 26 points at 1 m intervals across six north–south transects separated by 5 m within each replicate site (6 × 26 = 156 points per site). At each point, we used the plant growth forms ‘graminoid’, ‘herb’, ‘shrub’ and ‘tree’ to record the species richness and proportion of growth forms from ground to canopy, with differentiation at the species level whenever possible. Greenspace type classification was performed by calculating Shannon's diversity index on the woody plant (i.e., trees and shrubs) species, where sports fields had Shannon = 0 and nature parks had Shannon >0 (Figure S2), with greenspace types also showing distinct soil nutrient profiles (Table S1).

### 2.3 Soil sampling

Soil samples were collected between May 21 and 24 and June 11 and 14, 2022, from nine grid points at each 25 × 25 m site using an adapted Australian Microbiome Initiative sampling protocol (Bissett et al., 2016). A trowel decontaminated with ethanol and 5% Decon 90 (Decon Laboratories Ltd, Pennsylvania, USA) was used to collect approximately 50 g of soil from 0 to 5 cm depth at each grid point. Sterile nitrile gloves were worn during all sample collection steps to minimise contamination (Cando‐Dumancela et al., 2023). The soil samples were then pooled and homogenised in a sterile plastic bag. A 50 mL subsample of soil for DNA analysis was placed into a separate 50 mL sterile falcon tube and immediately put onto ice. Upon completion of field sampling activity, a ca.180 g subsample of each homogenised composite soil sample was placed into new bags and sent to CSBP Soil and Plant Analysis Laboratory (Bibra Lake, Western Australia) for analysis of 19 physical and chemical parameters, including pH, organic carbon, nitrate nitrogen and cation concentrations (see Table S2 for all parameter data). Soil moisture (%) was calculated in‐house using an oven‐drying process as follows: 40 g from each soil sample was transferred to an unsealed metal container, weighed and placed in an oven at 105°C for 24 h. Containers with the oven‐dried soils were then re‐weighed and the weight lost (= weight of water) as a percentage of total dry mass was calculated.

### 2.4 Soil short‐chain fatty acid sampling and quantification

No method was available to quantify short‐chain fatty acid concentrations in soils. Thus, we adapted a method from García‐Villalba et al. (2012), who examined short‐chain fatty acids in human faeces. This method used phosphoric acid for stabilising the short‐chain fatty acids, which for our purposes was preferable to snap‐freezing in liquid nitrogen due to long field days distant from the lab.

At each site, we collected soil from each grid point in the 25 × 25 m area using a decontaminated trowel (described above). The depth of soil collection was 3–5 cm. We used a scale in the field to weigh 1.1 g from each of eight site grid points and 1.2 g from one grid point to equal 10.0 g total soil weight per site and we placed these samples directly into a 50 mL tube pre‐filled with 16 mL of 0.5% phosphoric acid (Sigma Laboratory, Osterode am Harz, Germany). Each tube was then immediately placed on ice and stored in a −20°C laboratory freezer until short‐chain fatty acid extraction. To minimise the loss of volatiles, we collected short‐chain fatty acid soil sample at each grid point immediately prior to the soil sample designated for metagenomic and physicochemical analyses.

For short‐chain fatty acid extractions, sample tubes were removed from the freezer, thawed, vortexed for 20 s and centrifuged using a Sigma 3‐16KL centrifuge (Sigma Laboratory, Osterode am Harz, Germany) at 1000 relative centrifugal force (RCF). Then, to move the short‐chain fatty acids from the tube with phosphoric acid into the solvent ethyl acetate (100% hypergrade, Supelco Analytical, Bellefonte, PA, USA), 600 μL of supernatant was pipetted into a 2 mL polypropylene centrifuge tube with 600 μL of ethyl acetate. Each 2 mL tube was then vortexed for 20 s and centrifuged at 18 000 RCF on an Eppendorf Centrifuge 5425 (Eppendorf, Hamburg, Germany). Afterwards, 250 μL of the supernatant with the ethyl acetate was pipetted into 2 mL glass vials with 250 μL of the internal standard 4‐methylvaleric acid at 250 μM final concentration (Sigma‐Aldrich, St. Louis, MO, USA). During each step of the extraction process, the reagents were kept on ice to minimise the loss of volatile compounds. Standards were created with butyric acid (Sigma‐Aldrich, St. Louis, MO, USA), propionic acid (Sigma‐Aldrich, St. Louis, MO, USA) and acetic acid (Sigma‐Aldrich, St. Louis, MO, USA) diluted in ethyl acetate to 0.5, 1, 2, 5 and 10 μM concentrations each and the standards were stored in a −20°C freezer until gas chromatography analysis at Flinders Analytical (Flinders University).

The gas chromatography–mass spectrometry (GC–MS) set up included a SGE BP20 PEG WAX bromoform column (30 m × 0.25 mm × 0.25 μm; Trajan, Ringwood, VIC, Australia), fitted with an Agilent 7683 automatic liquid sampler autoinjector (G4513A), in tandem with an Agilent 7890 mass spectrometer (Agilent Technologies, Palo Alto, CA, USA). Samples were injected using a pulsed splitless injection of 1 uL. Column temperature was initially at 40°C, then increased to 250°C. Nitrogen was used as the carrier gas. A solvent delay was set at 2 min.

To create calibration curves, new standards were prepared on the day before GC–MS analysis and stored in a −20°C freezer until analysis (García‐Villalba et al., 2012). Calibration curves for butyric acid and propionic acid were obtained (*R* ^2^ = .998 and .994, respectively). Acetic acid was also quantified but could not achieve reliable calibration curves using our methods. After the calibration curves were created, the samples were injected with a hexane rinse and then again after every 10 samples. Data acquisition was performed using MassHunter Quantitative Analysis Software (Agilent Technologies, Palo Alto, CA, USA). Selected ion mass technique was chosen to quantify standard compounds with optimal sensitivity. The butyric acid peak with optimal height and shape was shown at *m/z* = 60 and this ion for butyrate was chosen for subsequent analyses.

### 2.5 Soil DNA extraction, PCR and sequencing

DNA extractions and quantifications were performed on the grid point‐pooled 50 g soil sub‐sample from each site in a dedicated DNA extraction laboratory at Flinders University. For soil DNA extractions, we used the Qiagen Power Soil kit (QIAGEN, Hilden, Germany) and followed the manufacturer's instructions. The extraction concentrations were then quantified using the Quantus fluorometer (Promega, Madison, WI, USA) and samples were sent to the South Australia Genomics Centre (Adelaide, South Australia) for library preparation via the Nextera XT DNA library prep kit for Illumina (Part No. FC131‐1024), protocol Version 05_05/19 and included 12 cycles of amplification. Libraries were all similar size and quantity. Equimolar pools were prepared and denaturing and on‐board clustering was performed using the MGI protocol. The 150 bp paired end read sequencing was completed using the MGI DNBSEQ‐G400 at South Australia Genomics Centre.

### 2.6 Bioinformatics

We performed quality control on the raw shotgun metagenomic sequence data of each sample using PRINSEQ++ (Cantu et al., 2019). Adapter sequences were removed using Cutadapt (Martin, 2011). Short read datasets were taxonomically classified using Kraken2 (Wood et al., 2019). Read sums were then normalised using R (Brame et al., 2024) by dividing by the expected genome lengths obtained from the NCBI (Nayfach & Pollard, 2015; Sayers et al., 2023) and then collated using Kraken2‐output‐manipulation. Next, relative abundance estimations were obtained using Bracken (Lu et al., 2017). The resulting data were filtered for butyrate‐producing bacteria using a list of 118 putative butyrate‐producing species derived from Vital et al. (2014) and NCBI using current classifications from Genome Taxonomy Database (see Table S3 for full butyrate producer list; Parks et al., 2021). Functional gene profiles were obtained using SUPER‐FOCUS (Silva et al., 2016), which reports the Seed subsystems (and corresponding functions) present in the datasets and profiles their abundances.

As our interest was on butyrate‐producing bacteria, reads were filtered in R (version 4.2.3; R Core Team, 2023) for annotations to the subsystem ‘Acetyl‐CoA fermentation to Butyrate’ and additionally for ‘tRNA aminoacylation, Phe’ for the gene *pheS* (EC_6.1.1.20), a single‐copy gene which we utilised for normalisation (Brame et al., 2022). The datasets were then filtered for the following terminal butyrate synthesis genes, based on Vital et al. (2014): Butyrate kinase (EC_2.7.2.7; gene *buk*), AcylCoA‐acetate CoA‐transferase, alpha subunit (EC_2.8.3.8; gene *butCoAT*) and Butyrate‐acetoacetate CoA‐transferase subunit_A (EC_2.8.3.9; gene *Ctf*). It is important to note that the enzymes EC_2.8.3.8 and EC_2.8.3.9 and the gene name *butCoAT* have many synonyms other than the ones listed above due to broad substrate specificity. Kraken2 classification data without the Bracken processing step was collated into a phyloseq object using the phyloseq package (version 1.42.0; McMurdie & Holmes, 2013) for differential abundance testing with Analysis of Compositions of Microbiomes with Bias Correction (ANCOMBC; version 2.0.3; Lin & Peddada, 2020).

### 2.7 Statistical analyses

All statistical analyses were performed in R (version 4.2.3; R Core Team, 2023), with statistical significance assessed at alpha = 0.05. Spatial maps were created using the ggmap package (version 3.0.2; Kahle & Wickham, 2013). Analysis of Compositions of Microbiomes with Bias Correction was done using the *ancombc2* function in the ANCOMBC package (version 2.0.3; Lin & Peddada, 2020) on the Kraken2 output data for differential abundance analyses. The ANCOMBC algorithm has been shown to minimise bias due to sampling fractions and reduces false discovery rates. The ggplot2 package (version 3.4.2; Wickham, 2016) was used for data visualisations. PERMANOVA (Adonis) tests and ordinations using principal coordinates analysis based on centred‐log‐ratio transformation were done using the vegan package (version 2.6.4; Oksanen et al., 2022). Random Forest regression modelling (Breiman, 2001) via the package ranger (Wright & Ziegler, 2015) was used to measure variable importance. The model fit was estimated using out‐of‐bag error from the bootstrap with mtry = 6, ntree = 500. The resulting Random Forest decision tree model explained 34.1% of the variance in our dataset. A plot of variable importance was created using random permutations for the value of each predictor variable in out‐of‐bag data, then calculating the mean decrease in node impurity.

## 3 RESULTS

### 3.1 Effects of greenspace type on soil butyrate‐producing bacterial species

**FIGURE 2:**
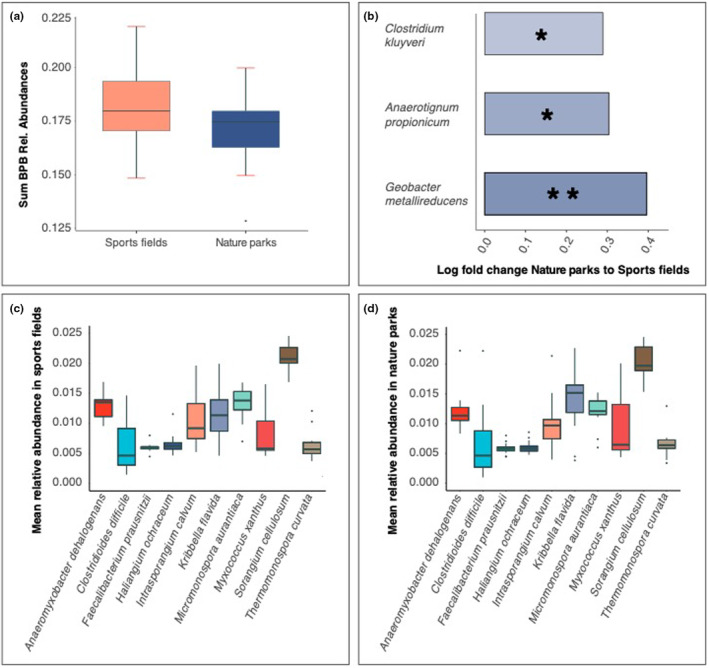
(a) Boxplots of total relative abundances of soil butyrate‐producing bacteria by greenspace type (not significantly different). Boxes show the median and interquartile range, while whiskers extend to the remaining range of data. (b) Log fold change (from nature parks to sports fields) in significantly differentially abundant soil butyrate‐producing bacterial species. Note these species shown have lower mean relative abundances than the 10 most common species highlighted in (c). (c, d) Boxplots showing the mean relative abundances of the 10 most abundant soil butyrate‐producing bacterial species by greenspace type. The *X*‐axis shows the 10 most common butyrate‐producing bacterial species. The *Y*‐axis shows the mean relative abundances. *p < .05, **p < .01.

Sports fields and nature parks had similar total relative abundances of butyrate‐producing bacteria (*t* = −1.096, df = 17, *p* = .29; Figure 2a). Sports fields had significantly higher relative abundances of three butyrate‐producing bacterial species: *Geobacter metallireducens* (lfc = 0.396, adj *p* = .004), *Anaerotignum propionicum* (lfc = 0.304, adj *p* = .043) and *Clostridium kluyveri* (lfc = 0.289, adj *p* = .024), compared to nature parks (Figure 2b). However, sports fields and nature parks shared the same 10 most abundant butyrate‐producing bacterial species (Figure 2c,d). In sports fields, the butyrate‐producing bacterial species with the two highest abundances were *Sorangium cellulosum* and *Micromonospora aurantiaca*. In nature parks, the butyrate‐producing bacterial species with the two highest abundances were *Sorangium cellulosum* and *Kribbella flavida*. Butyrate‐producing bacterial community compositions were also similar between sports fields and nature parks (Adonis PERMANOVA: *F* = 1.905, *R* ^2^ = .06, df = 1, *p* = .10; Figure S3).

### 3.2 Butyrate synthesis terminal genes in greenspace soils

**FIGURE 3:**
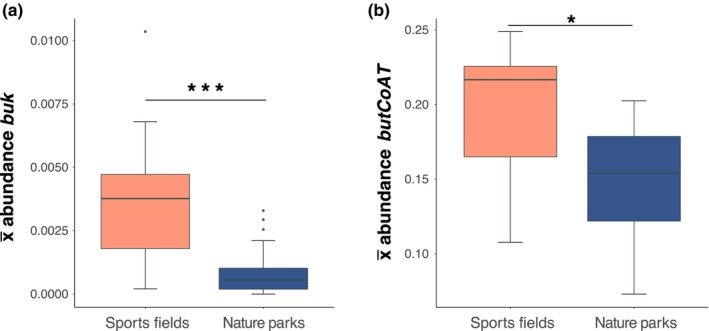
(a) Boxplots of abundances of butyrate metabolism genes *buk* by greenspace type (Wilcoxon test: *W* = 30, *p* < .001; sports fields *n* = 11, nature parks *n* = 21). (b) Boxplots of abundances of butyrate metabolism gene *butCoAT* by greenspace type (Welch's *t*‐test: *t* = −2.673, df = 16.3, *p* = .016). *Y*‐axis shows the mean count of the genes across the sites, normalised by the count of single copy gene *pheS*. Boxes show the median and interquartile range, while whiskers extend to the remaining range of data. *p < .05, ***p < .001.

Sports fields had a significantly higher abundance of the terminal butyrate production genes *buk* (Wilcoxon test: *W* = 30, *p* < .001; Figure 3a) and *butCoAT* (Welch's *t*‐test: *t* = −2.673, df = 16.3, *p* = .016; Figure 3b) than nature parks.

### 3.3 Butyrate concentrations in greenspace soils

**FIGURE 4:**
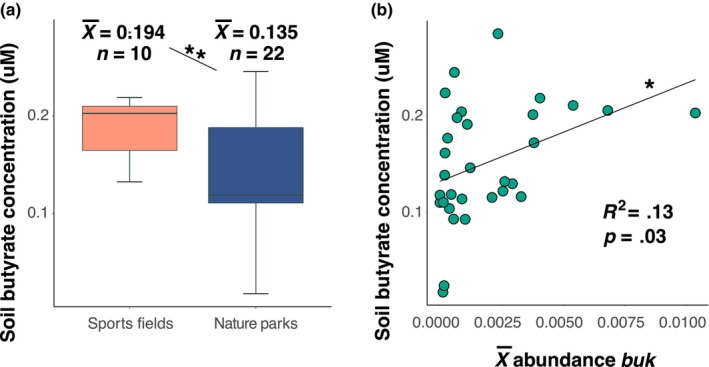
(a) Boxplots of soil butyrate concentrations by greenspace type (Welch's *t*‐test: *t* = −3.20, df = 22.9, *p* = .004), with sports fields having a higher concentration ($\bar{x}$ = 0.194 μM, *n* = 10) than nature parks ($\bar{x}$ = 0.135 μM, *n* = 22). The *Y*‐axis shows the soil butyrate concentration. Boxes show the median and interquartile range, while whiskers extend to the remaining range of data. (b) Relationship of the normalised abundance of butyrate metabolism gene *buk* with soil butyrate concentration (*F* = 5.295, df = 1 and 29, adj *R* ^2^ = .13, *p* = .029). The *X*‐axis shows the mean count of *buk* across the sites, normalised by the count of the single copy gene *pheS*. **p < .01.

Greenspace type had a strong effect on the soil butyrate concentration (Welch's *t*‐test: *t* = −3.20, df = 22.9, *p* = .004; Figure 4a), with sports fields having a higher concentration ($\bar{x}$ = 0.194 μM, *n* = 10) than nature parks ($\bar{x}$ = 0.135 μM, *n* = 22). Soil butyrate positively associated with *buk* read abundances (*F* = 5.295, df = 1 and 29, adj *R* ^2^ = .13, *p* = .029; Figure 4b), but not with *butCoAT* (*F* = 2.568, df = 1 and 29, adj *R* ^2^ = .05, *p* = .12). Soil butyrate also did not associate with the sum of butyrate‐producing bacterial read relative abundances (*F* = 0.14, df = 1 and 29, adj *R* ^2^ = −.03, *p* = .71). Interestingly, soil butyrate had no association with the gene *Ctf*, which encodes an enzyme that routes butyryl‐CoA into other non‐butyrate pathways (e.g., lysine degradation; *F* = 0.002, df = 1 and 29, adj *R* ^2^ = −.03, *p* = .97).

### 3.4 Effects of greenspace soil abiotic parameters on butyrate‐producing bacteria

**FIGURE 5:**
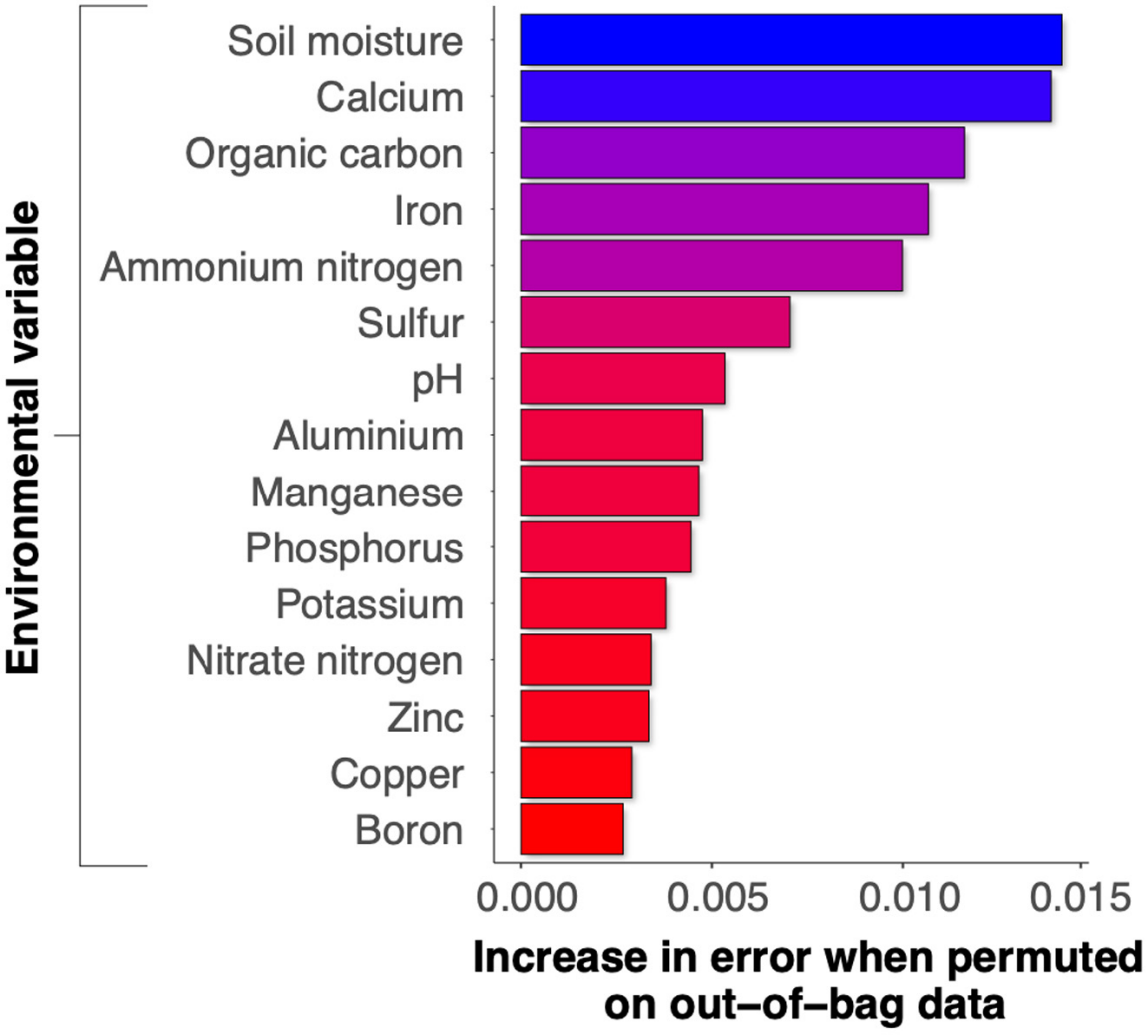
Random forest variable importance of soil physicochemical variables on soil butyrate concentration. Model performance *R* ^2^ = .34, out‐of‐bag MSE = 0.0024.

Firstly, we describe below our findings that associate soil abiotic parameters with soil butyrate concentrations. A Random Forest regression model with the soil parameters explained 33.3% of the variation in soil butyrate. Soil moisture (variable importance = 0.0116; Pearson's *r* = .50, *p* = .004), calcium (variable importance = 0.0111; Pearson's *r* = −.62, *p* < .001), iron (variable importance = 0.0110, Spearman *ρ* = .54, *p* = .002), ammonium nitrogen (variable importance = 0.0107; Spearman *ρ* = .58, *p* < .001) and organic carbon (variable importance = 0.009; Pearson's *r* = .45, *p* = .013) influenced soil butyrate concentration the most (Figure 5). Then we examined the associations of these five soil parameters to highlight potential effects on terminal butyrate production genes. Iron had a strong positive effect on *buk* read abundances (Spearman *ρ* = .56, *p* = .001) and calcium had a strong negative effect on *buk* read abundances (Spearman *ρ* = −.57, *p* = .001; Table 1). All other effects were negligible.

**TABLE 1:** Soil physicochemical values and correlation coefficients with soil butyrate concentrations and butyrate production terminal gene abundances.

Soil parameter	Sports fields (mean ± SD)	Nature parks (mean ± SD)	Correlation coefficient with soil butyrate conc.	Correlation coefficient with *buk*	Correlation coefficient with *butCoAT*
Moisture (%)	25.4 ± 5.40	18.9 ± 7.98	.50**	.15	.13
Calcium (meq/100 g)	11.2 ± 5.52	21 ± 7.53	−.62***	−.57**	−.28
Organic carbon (%)	4.05 ± 0.99	4.14 ± 0.38	.45*	.02	−.12
Iron (mg/kg)	117 ± 94.8	59.7 ± 46.9	.54**	.56**	.32
Ammonium nitrogen (mg/kg)	7.36 ± 2.20	5.91 ± 3.28	.58**	.12	.18

*Note*: Significance indicators: *p < .05, **p < .01 and ***p < .001.

## 4 DISCUSSION

We examined soils in two common urban greenspace types—sports fields and nature parks—and show that sports fields had higher butyrate concentrations and butyrate production gene abundances than nature parks. Soil abiotic conditions, specifically moisture, calcium, iron, ammonium nitrogen and organic carbon, strongly influenced soil butyrate concentrations and soil iron and calcium influenced soil butyrate production gene abundances. These findings suggest that urban greenspace management that impacts on these soil abiotic conditions (e.g., irrigation, fertiliser use, mowing) can influence the activity of butyrate producing bacteria. Our work shows that urban greenspaces, particularly sports fields, have the potential to provide exposure to health‐associated butyrate‐producing bacteria to humans. These findings have important implications to the designing and planning of urban greenspaces, plus to public health and human microbiome research.

### 4.1 Greenspace soil moisture affects the production of butyrate

We show that soil moisture levels strongly influenced greenspace soil butyrate concentrations. Butyrate production requires anaerobic conditions (Baxter et al., 2019) and wetting of soils (e.g., via rainfall, irrigation) can deplete oxygen and induce redox heterogeneity within soils, thereby establishing anoxic microsites with conditions conducive to butyrate production (Lacroix et al., 2023; Lentini et al., 2012). Our finding corroborates Brame et al. (2022) who found that hydrological fluctuations with wetting‐and‐drying cycles associated with greater butyrate‐producing bacterial read abundances in Australian soils. Thus, intermittent irrigation and/or rainfall events on urban greenspaces should provide the anoxic conditions required for fermentative butyrate synthesis and managing soil moisture via routine greenspace interventions (e.g., irrigation—in combination with sustainably managed plant cover and soil organic matter) could be a useful way to affect exposure of people to butyrate‐producing bacteria.

We derived a new method to directly quantify soil butyrate concentrations. This has not been done previously, likely because butyrate concentrations in soil tend to be low. Across our samples, we observed butyrate levels <100 μM, which is far lower than typically found in human gut samples (e.g., faecal butyrate concentrations tend to be 2–70 mM; Baxter et al., 2019; Kircher et al., 2022). Due to the volatility of butyrate, even with our methods that aimed to minimise butyrate loss (e.g., by obtaining each butyrate‐related soil sample before the bulk soil sample and by immediately fixing the soil sample in phosphoric acid), our measured butyrate concentrations may have been impacted by butyrate loss. Refining how butyrate is directly measured in soil would thus be useful.

### 4.2 Soil iron‐ and butyrate‐producing bacterial activity

We observed a strong positive association between greenspace soil iron levels and the abundances of the terminal butyrate production gene *buk*. The role of iron in the butyrate metabolic pathway is not fully understood and the reversibility of the enzyme butyrate kinase, encoded by the *buk* gene, suggests that a bidirectional relationship between iron and *buk* may be possible. This bidirectional relationship could be characterised as follows: (A) available iron facilitates butyrate production and, in reverse, (B) iron‐reducing bacteria oxidise butyrate and produce ferrous Fe(II) iron. We describe the evidence for these possible two explanations and discuss their implications below.

First, iron has been positively associated with measured butyrate concentrations. Dostal et al. (2015) showed that iron levels in faecal samples modulated butyrate‐related bacterial communities by using a polyfermenter model inoculated with the colonic microbiota from a child. Adjusting the iron levels (i.e., simulating different conditions within the proximal colon of a child) elicited substantial changes in the butyrate‐producing bacterial communities (e.g., Lachnospiraceae and Ruminococcaceae). However, the mechanisms of such an effect are not yet known. Furthermore, these authors observed that the abundances of the butyrate producer *Roseburia* were diminished by high levels of iron. *Roseburia* spp. utilise an energetically‐favourable CoA transferase with acetate to form butyrate (Hartmanis & Gatenbeck, 1984; Hillman et al., 2020). This CoA transfer employs a ping pong bi‐bi kinetic mechanism that bears double competitive substrate inhibition, where high concentrations of one substrate creates an inhibitory effect (Gheshlaghi et al., 2009).

On the other hand, our finding may reflect the role of iron as an electron acceptor in an anaerobic energy production pathway. Iron‐reducing bacteria can create energy by oxidising butyrate using the same enzymes from butyrate production, but in a reverse direction—they couple this oxidation with the reduction of iron from ferric Fe(III) to the more soluble ferrous Fe(II) state (Lentini et al., 2012). Indeed, coastal paddy soil research (Jiang et al., 2023) has showed that, under anoxic soil conditions, butyrate enhances the abundances of dissimilatory iron‐reducing bacteria, such as *Geobacter*, a genus we found in higher read abundances in sports fields.

Thus, the strong relationship between iron levels and butyrate‐producing bacterial activity appears to be bidirectional and non‐linear—it may vary depending on the particular concentration of iron. Future research could investigate the influence of varied iron concentrations and oxidation states, on butyrate‐producing bacteria in urban greenspace soils, with implications on landscape management practices that aim to modulate butyrate‐producing bacteria.

### 4.3 Abundances of buk and *butCoAT* genes could reflect the carbon substrate availability

We show that sports fields had higher abundances of the terminal genes *buk* and *butCoAT* than nature parks. The enzymes for both *buk* and *butCoAT* are reversible (Chang et al., 2021; Huang et al., 2000) and the direction of movement in their metabolic pathways could be related to soil conditions, such as carbon substrate availability. *Geobacter metallireducens* utilises the *buk* gene and had the highest differential abundance between sports fields and nature parks. *G. metallireducens* is known to grow vigorously when acetate is the only carbon source (Hartmanis & Gatenbeck, 1984; Lentini et al., 2012). *Anaerotignum propionicum* and *Clostridium kluyveri* utilise *butCoAT* rather than *buk* (Hillman et al., 2020) and also had higher read abundances in sports fields. *C. kluyveri* is a known butyrate producer and uses acetate and ethanol as its carbon sources (Seedorf et al., 2008). Therefore, acetate could be an important substrate for butyrate metabolism in sports field soils via both the *buk* and *butCoAT* metabolic pathways. This aligns with findings in Liddicoat et al. (2023), who reported increased potential metabolism of acetate in more highly disturbed (compared to more mature and natural) plant–soil systems. Though we did not evaluate acetate in this study, future research could further examine the role of acetate in shaping urban greenspace soil microbial communities.

### 4.4 Soil organic carbon and ammonium nitrogen associate with butyrate producing bacterial activity

We report that soil organic carbon and ammonium nitrogen correlated positively with soil butyrate concentrations. Carbon‐containing soil organic matter consists of plant, animal and microbial matter in varying stages of decomposition (Lal et al., 1997). In anoxic conditions, butyrate‐producing bacteria generate energy by fermenting organic carbon substrates into butyrate (Buckel, 2021). While increased organic carbon associated with enhanced butyrate production, we found similar levels of organic carbon between sports fields and nature parks, but higher levels of butyrate in sports fields. Thus, sports fields appear to have additional conditions that enhance the effects of organic carbon on butyrate producer activity. These findings show that greenspace management strategies that increase soil organic carbon alone may have limited effects on the activity of butyrate‐producing bacteria.

Ammonium nitrogen also associated positively with soil butyrate concentration. Nitrogen, together with carbon, is a key element present in soil organic matter, which acts as a store of nutrients, including cations such as ammonium. As such, ammonium nitrogen levels may reflect both nutrient storage (i.e., attached to soil organic matter) and cycling (i.e., from soil organic matter decomposition), such that higher ammonium nitrogen reflects increased fermentative pathways toward butyrate production. Alternately, increased soil ammonium nitrogen could reflect remnant levels from reduced ammonium breakdown. Soil ammonium is degraded by the feammox reaction. Jiang et al. (2023) observed that an increase in volatile fatty acids (e.g., butyrate) in coastal paddy soil from fertiliser degradation promoted the abundances of butyrate‐oxidising bacteria such as *Geobacter*. They proposed that iron reduction competes with the feammox reaction. In this way, an increase in butyrate oxidation could competitively inhibit the breakdown of ammonium, resulting in sustained levels of soil ammonium. Thus, future studies investigating how soil butyrate and ammonium are linked could clarify key roles of short‐chain fatty acids in plant–soil systems.

## 5 CONCLUSIONS

We show that urban greenspaces are reservoirs of butyrate‐producing bacteria, which were more actively producing butyrate in sports fields than nature parks. Soil conditions such as moisture, iron, ammonium nitrogen and organic carbon enhanced butyrate‐related activity, but we found evidence of bidirectional movement of enzymatic steps on those pathways. Commonly employed urban greenspace management practices (e.g., irrigation, fertiliser addition) therefore appear to play important roles in shaping soil butyrate‐producing bacterial activity. Our results suggest that sports fields could offer greater potential than nature parks to expose and supply health‐associated environmental butyrate‐producing bacteria to people. These findings will inform emerging opportunities for landscape designers, urban planners, ecologists and public health experts to work together to understand and modulate environmental microbial communities, with a focus on supporting human health via urban greenspace exposure.

## AUTHOR CONTRIBUTIONS

**Joel E. Brame:** Conceptualization (equal); data curation (lead); formal analysis (lead); funding acquisition (equal); investigation (lead); methodology (equal); project administration (lead); resources (equal); software (lead); visualization (supporting); writing – original draft (lead); writing – review and editing (lead). **Craig Liddicoat:** Conceptualization (equal); data curation (supporting); formal analysis (supporting); methodology (supporting); resources (supporting); software (equal); supervision (equal); visualization (supporting); writing – review and editing (equal). **Catherine A. Abbott:** Data curation (supporting); formal analysis (supporting); investigation (supporting); methodology (equal); project administration (supporting); resources (lead); supervision (supporting); writing – review and editing (equal). **Christian Cando‐Dumancela:** Conceptualization (supporting); data curation (supporting); investigation (supporting); methodology (supporting); resources (equal); validation (supporting); writing – review and editing (supporting). **Nicole W. Fickling:** Data curation (supporting); formal analysis (supporting); investigation (supporting); methodology (lead); writing – review and editing (supporting). **Jake M. Robinson:** Conceptualization (supporting); investigation (supporting); supervision (supporting); validation (supporting); visualization (equal); writing – review and editing (supporting). **Martin F. Breed:** Conceptualization (lead); data curation (supporting); formal analysis (supporting); funding acquisition (lead); investigation (equal); methodology (equal); project administration (supporting); resources (equal); supervision (lead); visualization (equal); writing – review and editing (equal).

## CONFLICT OF INTEREST STATEMENT

The authors have no conflicts of interest to declare that are relevant to the content of this article.

## BENEFIT‐SHARING STATEMENT

Benefits from this research accrue from the sharing of our data and results on public biological databases as described above.

## Supporting information


Appendix S1.

## Data Availability

The datasets generated during and/or analysed during the current study, as well as custom R code, are available at Figshare doi: 10.6084/m9.figshare.24993345. Metagenomic data has been made publicly available under PRJNA1066898 on Sequence Read Archive (SRA) at the National Center for Biotechnology Information (NCBI).
