# Supplementary material for: Urban sports fields support higher levels of soil butyrate and butyrate‐producing bacteria than urban nature parks
Source: Ecol Evol. 2024 Jul 22;14(7):e70057. doi: 10.1002/ece3.70057 (PMC11262829; doi:10.1002/ece3.70057)
Supplement: Supplementary file 1 — Appendix S1. [file ECE3-14-e70057-s001.docx]

**Supplementary Information**

*Urban sports fields support higher levels of soil butyrate and butyrate-producing bacteria than urban nature parks*

Joel E. Brame^a,b^, Craig Liddicoat^a,b^, Catherine A. Abbott^a^, Christian Cando-Dumancela^a,b^, Nicole W. Fickling, Jake M. Robinson^a,b^, Martin F. Breed^a,b^

^a^College of Science and Engineering, Flinders University, Bedford Park, SA 5042, Australia

^b^The Aerobiome Innovation and Research Hub (The AIR Hub), College of Science and Engineering, Flinders University, Bedford Park, SA 5042, Australia

**This PDF includes:**

Raw metagenomic sequence and preliminary annotation data

Table S1

Links for Tables S2 to S3

Figures S1 to S3

**Raw metagenomic read and preliminary annotation data**

We obtained 1,334,560,000 raw sequence reads from our soil samples, of which 84.76% had a Phred score >Q30. After quality control and removal of adapters, 1,179,992,332 paired reads remained. Taxonomic annotation of the reads identified the presence of 107 putative butyrate-producing bacterial species (= 90.7% of the target set of 118 putative butyrate producers; **Table S3**). This working list of 107 species was utilised in our taxonomic abundance analyses.

**Table S1**. Woody plant diversity and soil physicochemical parameters by land cover classification

|  | Sports fields | Parks |
| --- | --- | --- |
| Woody plant diversity | 0 | 1.260893 |
| Soil moisture | 25.38864 | 18.93182 |
| Ammonium nitrogen | 7.363636 | 5.909091 |
| Nitrate nitrogen | 20.54545 | 20.40909 |
| Phosphorus | 62.63636 | 33.86364 |
| Sulfur | 26.08182 | 14.93636 |
| Organic carbon | 4.047273 | 4.140909 |
| Conductivity | 0.2942727 | 0.2387727 |
| pH | 6.509091 | 6.468182 |
| Copper | 1.715455 | 1.672273 |
| Iron | 116.97273 | 59.72273 |
| Manganese | 8.062727 | 13.115455 |
| Zinc | 14.16727 | 20.98091 |
| Aluminium | 0.03636364 | 0.05954545 |
| Calcium | 11.19909 | 20.94136 |
| Magnesium | 4.663636 | 5.081364 |
| Potassium | 1.209091 | 1.415 |
| Sodium | 0.9754545 | 0.6231818 |
| Boron | 2.165455 | 2.984091 |

**Table S2**. Physicochemical parameters of soils by site.

See Figshare: doi: 10.6084/m9.figshare.24993345.

**Table S3**. Taxonomy of putative butyrate-producing bacteria.

See Figshare: doi: 10.6084/m9.figshare.24993345.


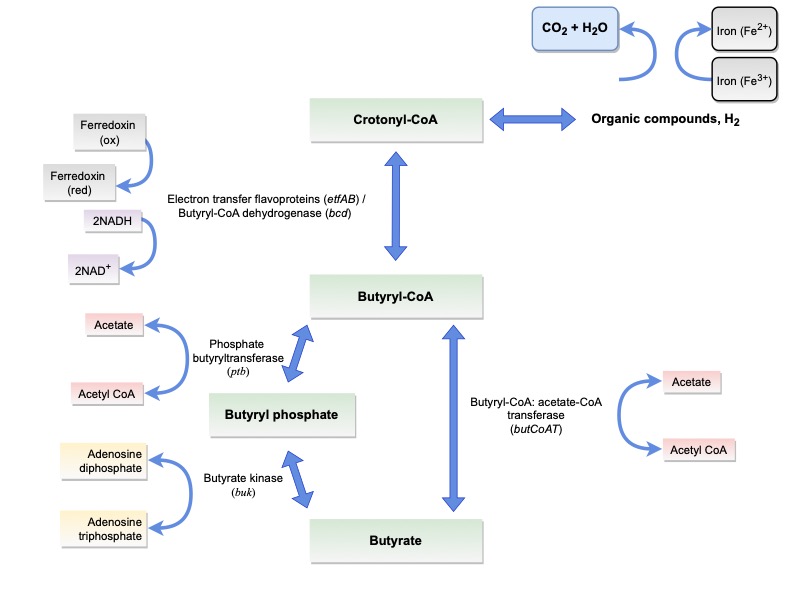


**Figure S1**. Biochemical pathway of butyrate production and butyrate oxidation.

**Figure S2**. Boxplots of woody plant species diversity by land cover. The y-axis shows the species diversity calculated by Shannon index. Boxes show the median and interquartile range, while whiskers extend to the remaining range of data.

**Figure S3**. Principal coordinates analysis based on Aitchison distances displaying showing similar soil butyrate-producing bacterial community composition between sports fields and nature parks (Adonis PERMANOVA: F = 1.905, R^2^ = 0.06, df = 1, *p* = 0.10).
